# Dibutyltin Dichloride Retards Leydig Cell Developmental Regeneration in Adult Rat Testis

**DOI:** 10.3389/fphar.2018.01320

**Published:** 2018-11-30

**Authors:** Xiande Huang, Taoye Ma, Yongsheng Yin

**Affiliations:** ^1^Department of Urology, Gansu Provincial Hospital, Lanzhou, China; ^2^Department of Urology, Second Provincial People’s Hospital of Gansu, Lanzhou, China

**Keywords:** dibutyltin dichloride, ethane dimethane sulfonate, leydig cell, developmental regeneration, testosterone

## Abstract

Dibutyltin dichloride (DBTCl), widely used as plastic stabilizer, can cause comprehensive toxicity. The present study aims to investigate the effects of DBTCl on rat Leydig cell developmental regeneration and characterize the related mechanism. Adult male Sprague Dawley rats were randomly divided into four groups and gavaged with saline (control) or 5, 10, or 20 mg/kg/day of DBTCl consecutively for 10 days. At the end of the DBTCl treatment, all rats received a single intraperitoneal injection (i.p.,) of 75 mg/kg ethane dimethane sulfonate (EDS) to eliminate all the adult Leydig cells and to induce Leydig cell developmental regeneration. Leydig cell developmental regeneration was evaluated by measuring the levels of serum testosterone, luteinizing hormone, and follicle-stimulating hormone on days 7, 35, and 56 post-EDS. Leydig cell gene and protein expression levels, as well as cell morphology and cell counts were also carried out on day 56 post-EDS. The present study found that DBTCl significantly reduced serum testosterone levels on days 35 and 56 post-EDS, but increased serum luteinizing hormone (LH) and follicle-stimulating hormone (FSH) levels on day 56 at ≥ 5 mg/kg/day. The mRNA and protein levels of Leydig (*Lhcgr, Scarb1, Star, Cyp11a1, Hsd17b3*, and *Hsd11b1*) and Sertoli cells (*Fshr, Amh*, and *Sox9*) were significantly downregulated in the DBTCl-treated testes compared to the control. Immunohistochemical staining showed that DBTCl-treatment caused fewer regenerated Leydig cells and impaired Sertoli cell development and function in the testis on day 56 post-EDS. In conclusion, the present study demonstrates that DBTCl retards rat Leydig cell developmental regeneration by downregulating steroidogenesis-related enzymes at the gene and protein levels, inhibiting Leydig cell proliferation and impairing Sertoli cell function and development.

## Introduction

Organotins are widely used in agricultural biocides, wood preservatives, and antifouling paints for boats and some other industrial applications. Every year, large amounts of organotins are released into the environment, and they have become worldwide environmental pollutants. Dibutyltin (DBT) is one of the organotins primarily used for stabilizing polyvinyl chloride plastic products, as it prevents the degradation of plastics by combining with the hydrochloric acid (HCl) liberated from the polymer, which produces dibutyltin dichloride (DBTCl) (Boyer, 1989). Measurable levels of DBTCl has so far been found in drinking water (Forsyth et al., 1993), house dust (Fromme et al., 2005), foodstuffs (Hong et al., 2002), and surface waters (Bancon-Montigny et al., 2004). In fact, DBTCl is readily detected in human blood and liver samples (Nielsen and Strand, 2002; Brown et al., 2017) and has been shown to cause extensive toxicities to animals, including placental and fetal toxicity, immune toxicity, and endocrine toxicity (Brown et al., 2017; Furukawa et al., 2017; Milton et al., 2017; Zhang et al., 2018).

Leydig cells in the testis interstitial compartment contribute 95–99% of circulatory testosterone (Wu et al., 2017). Testosterone biosynthesis is an enzyme-controlled steroidogenic process. Extracellular cholesterol is transported into the mitochondria by transporters called cell membrane scavenger receptor class B type 1 (SCARB1, encoded by *Scarb1*) and steroidogenic acute regulatory protein (STAR, encoded by *Star*), where it is catalyzed into testosterone by enzymes including cholesterol side-chain cleavage enzyme (CYP11A1, encoded by *Cyp11a1*), 3β-hydroxysteroid dehydrogenase 1 (3β-HSD1, encoded by *Hsd3b1*), cytochrome P450 17α-hydroxylase/17,20-lyase (CYP17A1, encoded by *Cyp17a1*), and 17β-hydroxysteroid dehydrogenase 3 (17β-HSD3, encoded by *Hsd17b3*). Among these enzymes, STAR and CYP11A1 are the rate-limiting ones (Wang et al., 2011). Leydig cell differentiation and development are regulated by endocrine system. Gonadotrophs in the pituitary gland secrete luteinizing hormone (LH, LH-β subunit is encoded by *Lhb*) (Zhang et al., 2018). LH promotes Leydig cell development and androgen production by directly binding to luteinizing hormone/chorionic gonadotropin receptor (LHCGR) on its surface (Melsert et al., 1991), leading to an increase in steroidogenic enzyme activity, particularly steroidogenic enzyme activities of rate-limiting STAR and CYP11A1, through cAMP-PKA signaling pathway (Foster, 1996).

Sertoli cell is known as Leydig-supporting cell. Another hormone secreted by gonadotrophs, called follicle-stimulating hormone (FSH, FSH-β subunit is encoded by *Fshb*), binds to follicle-stimulating hormone receptor (FSHR, encoded by *Fshr*) on Sertoli cells to stimulate desert hedgehog (DHH, encoded by *Dhh*) and anti-Müllerian hormone (AMH, encoded by *Amh*) expression and secretion (Kim and Griswold, 2001; Blagosklonova et al., 2002), which promotes Leydig cell development and regeneration (Sriraman et al., 2001; O’Shaughnessy et al., 2008). For Sertoli cells themselves, SOX9 serves not only as a biomarker (Koopman, 1999) but also as a critical developmental and maturation transcription factor (Foster, 1996; Barrionuevo et al., 2006).

Although comprehensive DBTCl toxicities have been demonstrated (Brown et al., 2017; Furukawa et al., 2017; Milton et al., 2017; Zhang et al., 2018), no available studies have described the effects of DBTCl on Leydig cell development. Herein, this study aims to address the effects of DBTCl on Leydig cell developmental regeneration and the related mechanism by adopting an ethane dimethane sulfonate (EDS)-induced rat Leydig cell regeneration model.

## Materials and Methods

### Chemicals

DBTCl was purchased from Sigma-Aldrich (St. Louis, MO, United States). Immulite2000 Total Testosterone Kit was purchased from Sinopharm Group Medical Supply Chain Services Co., Ltd. (Hangzhou, Zhejiang, China). Ethane dimethane sulfonate was purchased from Pterosaur Biotech (Hangzhou, China). Trizol was purchased from Invitrogen (Carlsbad, CA, United States). SYBR Green qPCR Kit and BCA Protein Assay Kit were purchased from Takara (Otsu, Japan). RIPA buffer was purchased from Bocai Biotechnology (Shanghai, China).

### Animal Administration

Seventy-two 51-day-old male Sprague-Dawley rats (Laboratory Animal Center of Lanzhou University, Lanzhou, China) were raised in a 12 h dark/light cycle temperature at 23 ± 2°C and relative humidity of 45–55%. Free access to water and food was provided. Once the rats got adjusted to the new environment they were randomly divided into four groups (18 animals per group). The rats were kept in IVC cages (three rats per cage) on soft chip bedding and provided pellet chow (Shanghai Laboratory Animal Center). DBTCl was dissolved in saline and gavaged to rats. Rats in group 1 gavaged saline served as the control, while rats in groups 2, 3, and 4 received DBTCl at doses of 5, 10, and 20 mg/kg/day, respectively. The doses were selected with reference to previous studies (Ema et al., 1992; Furukawa et al., 2017). The treatments were administered consecutively for 10 days. The body weight of each rat was recorded every 2 days.

EDS was dissolved in a mixture of dimethyl sulfoxide and deionized sterile water (1:3, v/v). At the end of the 10-day DBTCl gavaging, all rats received a single i.p., of 75 mg/kg EDS to eliminate all Leydig cells. Rats (six animals from each group) were sacrificed on days 7, 35, and 56 post-EDS treatment by asphyxiation with CO_2_. Trunk blood was collected, placed in a gel glass tube, and centrifuged at 1500 × g for 10 min to collect serum samples, which were stored at -80°C for hormone analysis. Additionally, each pair of testes was separated and weighted. One testis from each animal was frozen in liquid nitrogen and stored at -80°C for subsequent gene and protein expression level analysis. The contralateral testis was punctured thrice using a G27 needle and then fixed in Bouin’s solution for immunohistochemical analysis. All experimental procedures were performed in strict accordance with the international regulation on animal welfare and approved by the Lanzhou University’s Animal Care and Use Committee.

### Testosterone Measurement

Serum testosterone concentration was measured using Immulite2000 Total Testosterone Kit according to manufacturer’s instructions (Siemens, Germany), as previously described (Ge and Hardy, 1998). The minimal detection limit of testosterone was 0.2 pg/ml. The internal control contained 100 pg/ml testosterone dissolved in the same culture media. Inter-assay variation was within 10%.

### ELISA for Serum LH and FSH Levels

The serum LH and FSH levels were measured using enzyme-linked immunosorbent assay (ELISA) kit according to the manufacturer’s instructions (Chemicon, CA, United States). First, 200 μL samples and 50 μL assay diluent were added to pre-coated 96-well plates. The plates were then incubated for 2 h at room temperature and washed 5 times with washing buffer. A total of 100 μL peroxidase-conjugated IgG anti-LH or anti-FSH solution was added into each well and incubated at room temperature for another 2 h. The plates were again washed 5 times with washing buffer. Next, 100 μL of substrate buffer was added into each well and incubated in the dark for 30 min at room temperature. The enzyme reaction was stopped by adding 50 μL stop solution. Finally, the quantification of LH and FSH levels were obtained on a microplate reader at 550 nm with correction wavelength at 450 nm. For LH, the intra-/inter-assay coefficients of variation were 6 and 8%, respectively. And for FSH, the coefficients were 4 and 7%, respectively.

### RNA Isolation and Real-Time PCR (qPCR)

Total RNAs were isolated and purified from the testes using Trizol (Invitrogen, CA, United States). After isolation and purification by DNase, the concentrations of total RNAs were determined by reading the optical density (OD) value at 260 nm. The RNAs were reversely transcribed into cDNAs using random hexamers and MMLV reverse transcriptase in the Reverse-Transcription reagent kit (Invitrogen, United States). The cDNAs were then subjected to qPCR with SYBR Green qPCR Kit (Takara, Otsu, Japan) to quantify the gene expression levels of *Lhcgr, Scarb1, Star, Cyp11a1, Hsd3b1, Cyp17a1*, and *Hsd17b3* in Leydig cells and *Fshr, Amh, Dhh*, and *Sox9* in Sertoli cells. The reaction mixture included 10 μL SYBR Green Mix, 1 μL forward primers, 1 μL reverse primers, 1 μg diluted cDNA, and 3–6 μL ddH2O. The reaction cycle was performed as follows: 95°C for 2 min, followed by 40 cycles of 95°C for 10 s and 59°C for 30 s. Fluorescence was detected on a Bio-Rad qPCR system (Bio-Rad Laboratories, Inc., Hercules, CA, United States). The Ct values were read and the data were processed using absolute quantification and standard curve methods, as previously described (Ge et al., 2005), except that β-Actin (*Actb*) was adopted in this study as the internal control. The melting curve was examined for the quality of PCR amplification for each sample. Primer information is listed in Supplementary Table 1.

### Western Blot on Total Testis Protein Samples

Testis samples were homogenized in ice and lysed in a 0.5–1 ml mixture of RIPA (Bocai Biotechnology, Shanghai, China) and protease inhibitor (Amyjet Scientific Inc, China) (1000:1) after being washed twice with PBS. The lysate was centrifuged at 12,000 × g for 15 min at 4°C. After centrifugation, the supernatant was collected and the protein concentration was determined spectrophotometrically by absorbance reading at 595 nm, using BCA Assay Kit (Takara, United States), according to the manufacturer’s instructions. Supernatant containing 20 μg protein was mixed with 5 × loading buffer and H_2_O. The mixture was boiled at 100°C for 10 min. SDS polyacrylamide gel electrophoresis was performed at a constant voltage of 80–120 volts, after which the protein in the gel was electrophoretically transferred onto nitrocellulose membranes. After blocking the non-specific binding by immersing the membranes in 5% non-fat milk at room temperature for 30 min, the membranes were incubated overnight at 4°C with primary antibodies against the following antigens: LHCGR, SCARB1, STAR, CYP11A1, 3β-HSD1, CYP17A1, 17β-HSD3, and 11β-HSD1 in Leydig cells, and FSHR, AMH, DHH, and SOX9 in Sertoli cells. The membranes were further incubated with horseradish peroxidase-conjugated secondary antibody (1:5000, BioWorld, MN, United States) at room temperature for 2 h. The membranes were washed thrice, before and after incubation, with tris-buffered saline containing tween 20. Finally, the protein was visualized by exposing the strips to enhanced chemiluminescence solution (Pierce Chemical Co, IL, United States). The protein levels were quantified by analyzing the gray levels of bands through Image Lab software and normalized to ACTB, the internal control. Antibody information is listed in Supplementary Table 2.

### Immunohistochemical Staining and Cell Counting

3β-HSD1 and SOX9 were used as the biomarkers of Leydig cells and Sertoli cells, respectively (Koopman, 1999; Guo et al., 2013). To enumerate the number of 3β-HSD1-positive Leydig cells and SOX9-positive Sertoli cells, the testes were subjected to the fractionator technique, as previously described (Mendis-Handagama et al., 1989). Six testes per group were randomly selected at each time point. Each testis was cut into eight disks, from which two disks were randomly selected. The two disks were then further cut into four pieces, from which one piece was randomly selected. The pieces per testis were embedded in paraffin in a tissue array and sectioned into 6-μm-thick sections. Approximately ten sections were randomly selected from each testis per rat. The sections were used for immunohistochemical staining. Avidin-biotin immunohistochemical staining was performed according to the manufacturer’s instructions (Vector, Burlingame, CA, United States). Microwave heating in a citrate buffer (10 mM, pH 6.0) for 10 min was performed for antigen retrieval. The endogenous peroxidase was blocked with 0.5% H_2_O_2_ in methanol for 30 min. The sections were incubated in 3β-HSD1 or SOX9 antibody (1:1000 dilution, v/v) for 1 h at room temperature. The antibody-antigen complexes were visualized with diaminobenzidine as the brown cytoplasmic staining for positively labeled Leydig cells and the brown nuclear staining for positively labeled Sertoli cells. The sections were counterstained with Mayer hematoxylin, dehydrated in graded concentrations of alcohol, and cover-slipped with resin. Images were taken and total microscopic fields per section were counted. The total number of Leydig cells was calculated by multiplying the number of Leydig or Sertoli cells counted in a known fraction of the testis by the inverse of the sampling probability.

### Computer-Assisted Image Analysis of Cell Size and Nuclear Size

Leydig cells were identified by 3β-HSD1 staining as described above. The cell size, nuclear size, and cytoplasmic size of Leydig cells were calculated as previously described (Liu et al., 2016). Five randomly selected fields in each of three non-adjacent sections per testis were captured using a BX53 Olympus microscope (Tokyo, Japan) equipped with a digital camera interfaced to a computer. The images that were displayed on the monitor represented a partial area of a testis. Cell size, nuclear size, and cytoplasmic size were estimated using the image analysis software (Image-Pro Plus; Media Cybernetics, Silver Spring, MD, United States). More than 50 Leydig cells were evaluated in each testis. The cell size and nuclear size were calculated as μm^3^ and the cytoplasmic size was calculated by subtracting nuclear size from cell size.

### Semi-Quantitative Immunohistochemical Measurement of 11β-HSD1 and SOX9 Densities

11β-HSD1 is a marker protein for Leydig cells (Ge and Hardy, 1998). SOX9 is a transcription factor uniquely expressed in Sertoli cells for its function (Koopman, 1999). Immunohistochemical staining of 3β-HSD1 and SOX9 was performed as stated above. Target protein density and background area density were measured using the image analysis software (Image-Pro Plus; Media Cybernetics, Silver Spring, MD, United States) according to the manufacturer’s instructions. More than 50 Leydig or Sertoli cells were evaluated in each testis, and the density of each sample was averaged.

### Statistical Analysis

All data are presented as the mean ± standard errors (SEM). The statistical significance was analyzed using one-way ANOVA followed by *ad hoc* Turkey’s multiple comparisons to the control. Statistical analysis was performed using GraphPad Prism (version 6, GraphPad Software Inc., San Diego, CA, United States), and *p* < 0.05 was considered statistically significant.

## Results

### General Toxicity of DBTCl

To analyze the general toxicity of DBTCl, rat body weights were recorded before and after DBTCl treatment as well as on days 7, 35, and 56 post-EDS treatment (Table 1). Rat testis weights were recorded on days 7, 35, and 56 post-EDS treatment (Table 1). DBTCl significantly decreased the body weights at 20 mg/kg on day 35, and at ≥ 10 mg/kg on day 56. These results were in agreement with previous studies (Harazono and Ema, 2003; Furukawa et al., 2017). Testis weights were significantly decreased after DBTCl treatment at ≥ 10 mg/kg on day 56. No mortalities were observed among the rats.

**Table 1:** Body and testis weight.

Parameters	Dosage (mg/kg)			
	**0**	**5**	**10**	**20**
	
**Body weight (g)**				
Before DBTCl treatment	300.35 ± 10.33	305.47 ± 3.45	293.23 ± 5.23	298.23 ± 13.45
After DBTCl treatment	325.43 ± 15.12	330.23 ± 30.23	280.68 ± 43.85	285.85 ± 50.24
Post-EDS day 7	310.23 ± 19.43	295.53 ± 20.56	315.25 ± 43.52	307.84 ± 40.23
Post-EDS day 35	389.95 ± 25.23	350.23 ± 23.64	345.98 ± 52.89	322.23 ± 35.53^∗^
Post-EDS day 56	532.23 ± 30.12	495.32 ± 32.73	410.23 ± 37.54^∗∗∗^	395.03 ± 56.23^∗∗∗^
**Testis weight (g)**				
Post-EDS day 7	1.30 ± 0.53	1.34 ± 0.68	1.27 ± 0.72	1.25 ± 0.23
Post-EDS day 35	1.41 ± 0.78	1.40 ± 0.59	1.35 ± 0.17	1.30 ± 0.20
Post-EDS day 56	2.46 ± 0.19	2.12 ± 0.24	1.83 ± 0.25^∗^	1.38 ± 0.35^∗∗∗^


*Mean ± SEM, *n* = 6. ^∗^, ^∗∗^, ^∗∗∗^ indicate significant difference when compared to the control (CON) at *p* < 0.05, 0.01, and 0.001 at each time point, respectively.*

### The Effects of DBTCl on Hormone Levels

**FIGURE 1:**
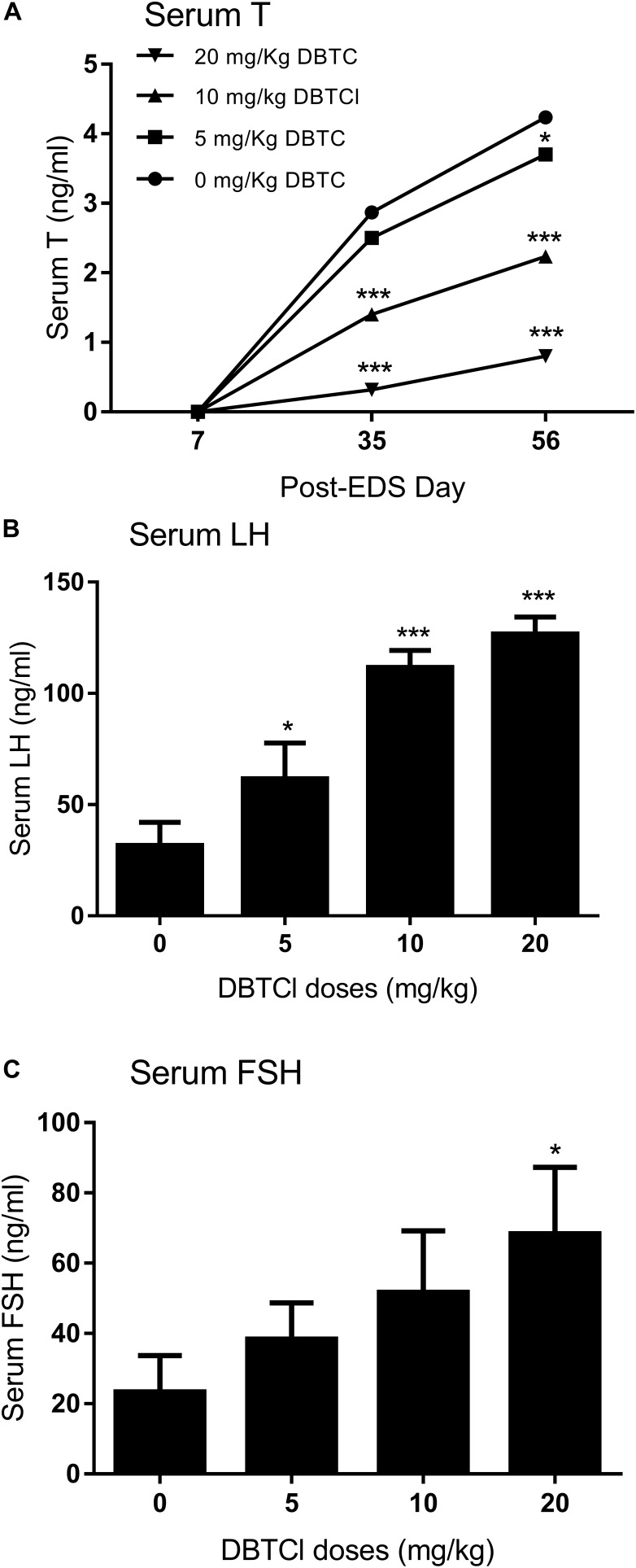
Serum levels of testosterone (T), luteinizing hormone (LH), and follicle-stimulating hormone (FSH) after dibutyltin dichloride (DBTCl) treatment. **(A)** Serum T levels on post-EDS (ethane dimethane sulfonate) days 7, 35, and 56. **(B)** Serum LH levels on post-EDS day 56. **(C)** Serum FSH levels on post-EDS day 56. Mean ± SEM, *n* = 6. ^∗^, ^∗∗^, ^∗∗∗^ indicate significant difference when compared to the control at *p* < 0.05, 0.01, and 0.001, respectively.

Sera were collected on days 7, 35, and 56 post-EDS treatment for quantification of testosterone, LH, and FSH levels (Figure 1). Serum testosterone levels in all groups were undetectable on day 7, indicating that all Leydig cells were completely eliminated by EDS. Testosterone levels in all groups gradually increased on days 35 and 56, implying the regeneration of Leydig cell in the testis. A DBTCl dose-dependent decrease in testosterone levels at ≥ 10 mg/kg was seen on days 35 and 56 (Figure 1A), which suggests that Leydig cell developmental regeneration was retarded by DBTCl. DBTCl also significantly increased serum LH levels at ≥ 5 mg/kg and FSH levels at 20 mg/kg on day 56 post-EDS (Figures 1B,C), which suggests that both Leydig cell and Sertoli cell functions are impaired by DBTCl.

### The Effects of DBTCl on Gene Expression Levels of Leydig and Sertoli Cells

**FIGURE 2:**
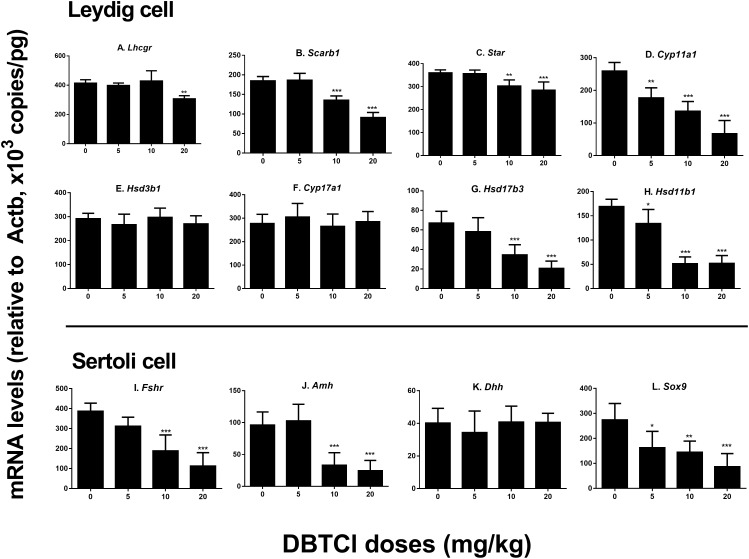
Expression levels of Leydig and Sertoli cell genes in the testes of dibutyltin dichloride (DBTCl)-treated rats on the post-EDS day 56. Leydig cell genes: **(A)** *Lhcgr*, **(B)** *Scarb1*, **(C)** *Star*, **(D)** *Cyp11a1*, **(E)** *Hsd3b1*, **(F)** *Cyp17a1*, **(G)** *Hsd17b3*, and **(H)** *Hsd11b1*. Sertoli cell genes: **(I)** *Fshr*, **(J)** *Amh*, **(K)** *Dhh*, and **(L)** *Sox9*. Mean ± SEM, *n* = 6. ^∗^, ^∗∗^, ^∗∗∗^ indicate significant difference when compared to the control (0 mg/kg DBTCl) at *p* < 0.05, 0.01, and 0.001, respectively.

We conducted qPCR experiment to measure the gene expression levels of *Lhcgr, Scarb1, Star, Cyp11a1, Hsd3b1, Cyp17a1, Hsd17b3*, and *Hsd11b1* in Leydig cells and *Fshr, Amh, Dhh*, and *Sox9* in Sertoli cells on day 56 (Figure 2). It was found that among the Leydig cell genes, DBTCl significantly downregulated *Cyp11a1* and *Hsd11b1* levels at ≥ 5 mg/kg (Figures 2D,H), downregulated *Scarb1, Star*, and *Hsd17b3* levels at ≥ 10 mg/kg (Figures 2B,C,G), and downregulated *Lhcgr* levels at 20 mg/kg (Figure 2A). No effects on *Hsd3b1* and *Cyp17a1* levels were seen even when tested at the highest dose (Figures 2E,F). Among the Sertoli cell genes, DBTCl significantly downregulated *Sox9* levels at ≥ 5 mg/kg (Figure 2L) and downregulated *Fshr* and *Amh* levels at ≥ 10 mg/kg (Figures 2I,J) but had no effects on *Dhh* levels even when tested at the highest dose (Figure 2K). These results suggest that DBTCl selectively exerts negative effects on Leydig and Sertoli cell gene expressions.

### The Effects of DBTCl on Protein Expression Levels of Leydig and Sertoli Cells

**FIGURE 3:**
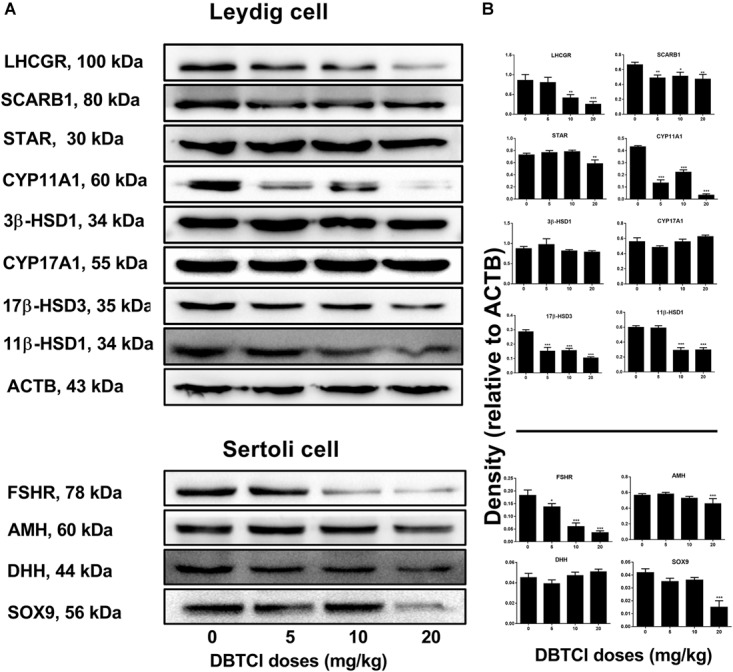
Expression levels of Leydig and Sertoli cell proteins in the testes of dibutyltin dichloride (DBTCl)-treated rats on the post-EDS day 56. **(A)** Gel images. **(B)** Quantitative results. Leydig cell proteins: LHCGR, SCARB1, STAR, CYP11A1, 3β-HSD31, CYP17A1, 17β-HSD3, and 11β-HSD1. Sertoli cell proteins: FSHR, AMH, DHH, and SOX9. Mean ± SEM, *n* = 3. ^∗^, ^∗∗^, ^∗∗∗^ indicate significant difference when compared to the control (0 mg/kg DBTCl) at *p* < 0.05, 0.01, and 0.001, respectively.

Western blot experiments were conducted to measure the protein expression levels of LHCGR, SCARB1, STAR, CYP11A1, 3β-HSD1, CYP17A1, 17β-HSD3, and 11β-HSD1 in Leydig cells and FSHR, AMH, DHH, and SOX9 in Sertoli cells on day 56 post-EDS treatment (Figure 3). It was found that protein levels in both Leydig and Sertoli cells followed a similar trend with respect to the changes in gene expression levels. As shown in Figure 3, among the Leydig cell proteins, DBTCl significantly downregulated SCARB1, CYP11A1, and 17β-HSD3 levels at ≥ 5 mg/kg, downregulated LHCGR and 11β-HSD1 at ≥ 10 mg/kg, and downregulated *Star* levels at 20 mg/kg but had no effects on *3β-HSD1* and *CYP17A1* levels even when tested at the highest dose. As shown in Figure 3, among the Sertoli cell proteins, DBTCl significantly downregulated FSHR levels at ≥ 5 mg/kg, downregulated AMH and SOX9 levels at 20 mg/kg, but it had no effects on DHH levels even when tested at the highest dose. These results suggest that DBTCl selectively exerts negative effects on protein expression in Leydig and Sertoli cells.

### The Effects of DBTCl on Leydig Cell Number and 11β-HSD1 Density

**FIGURE 4:**
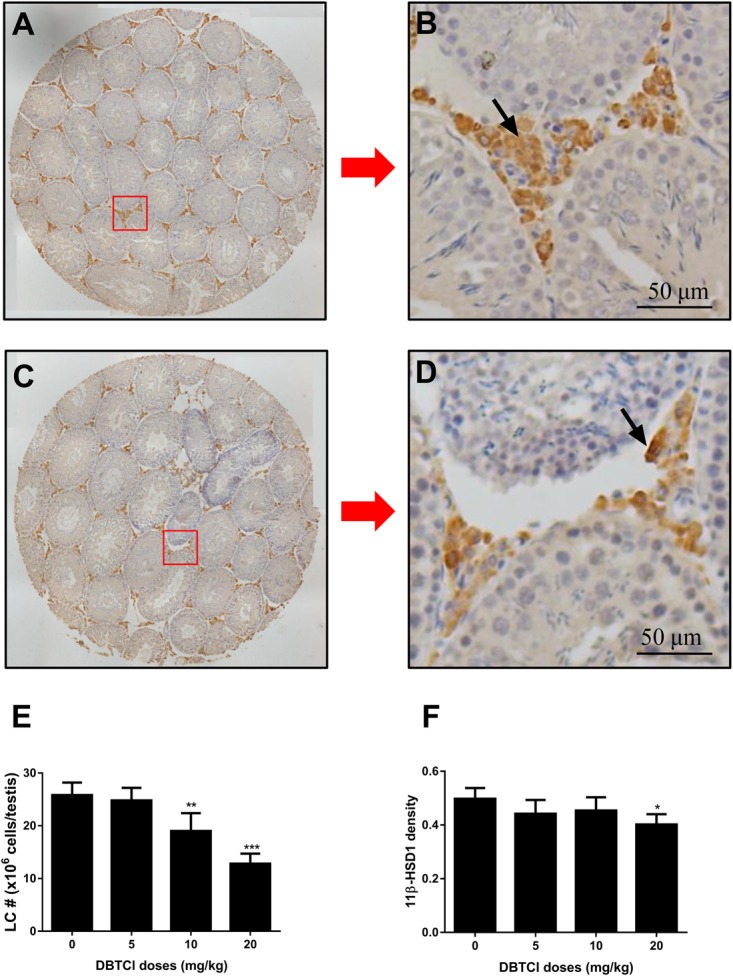
The effects of dibutyltin dichloride (DBTCl) on Leydig cell (LC) number and maturation in rat testes on post-EDS day 56. Leydig cells were identified by immunohistochemical staining of 11β-HSD1 and LC number was enumerated by the stereological method. 11β-HSD1 densities were determined by semi-quantitative assay. **(A**,**B)** for control group. **(C**,**D)** for 20.0 mg/kg DBTCl group. Bar = 50 μm. Black arrow points to Leydig cells. **(E)** Quantification of LC number (#). **(F)** Semi-quantitative assay of 11β-HSD1 densities. Mean ± SEM, *n* = 6. ^∗^, ^∗∗^, ^∗∗∗^ indicate significant difference when compared to the control group at *p* < 0.05, 0.01, and 0.001, respectively.

Leydig cells were stained by its advanced-stage biomarker 11β-HSD1 (Guo et al., 2013). As shown in Figure 4, DBTCl treatment significantly reduced 11β-HSD1-positive cells in rat testis at ≥ 10 mg/kg on day 56 post-EDS treatment. Further analysis showed that DBTCl decreased 11β-HSD1 densities in individual positive-staining cells at 20 mg/kg. These results indicate that DBTCl retards the Leydig cell developmental regeneration process.

### The Effects of DBTCl on Sertoli Cell Number and SOX9 Density

**FIGURE 5:**
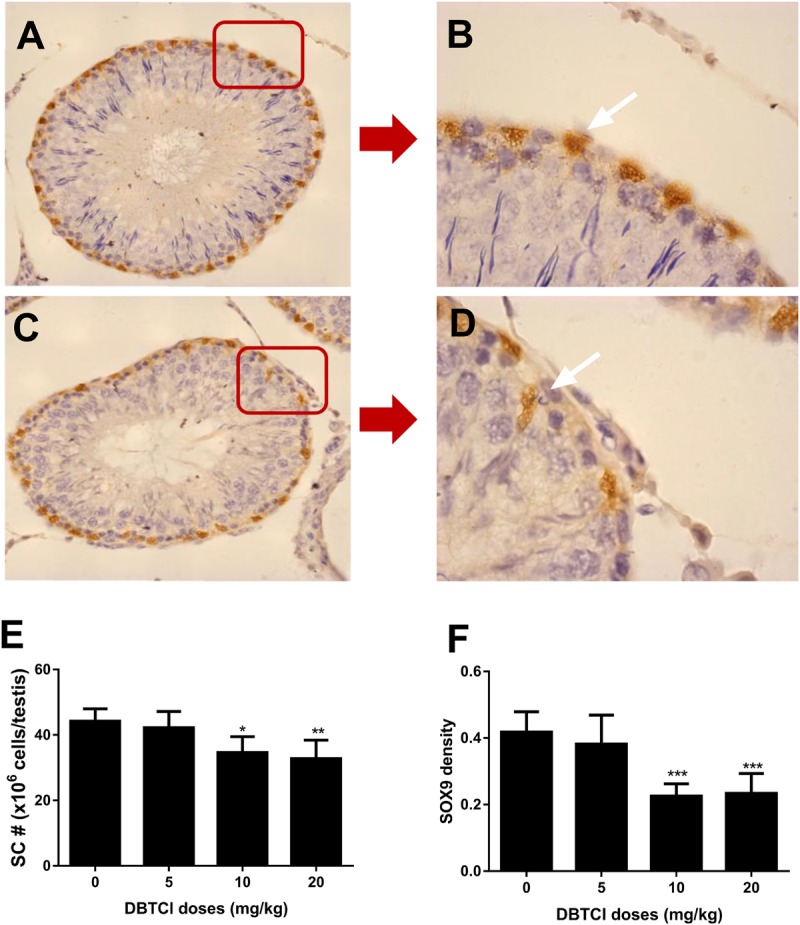
The effects of dibutyltin dichloride (DBTCl) on Sertoli cell (SC) number and maturation in rat testes on post-EDS day 56. Sertoli cells were identified by immunohistochemical staining of SOX9 and SC number was enumerated by the stereological method. SOX9 densities were determined by semi-quantitative assay. **(A**,**B)** for control group. **(C**,**D)** for 20.0 mg/kg DBTCl group. Bar = 50 μm. White arrow points to Sertoli cells. **(E)** Quantification of LC number (#). **(F)** Semi-quantitative assay of SOX9 densities. Mean ± SEM, *n* = 6. ^∗^, ^∗∗^, ^∗∗∗^ indicate significant difference when compared to the control group at *p* < 0.05, 0.01, and 0.001, respectively.

Sertoli cells were identified by staining with biomarker SOX9 (Koopman, 1999), which also functions as a transcription factor for development and maturation (Foster, 1996; Barrionuevo et al., 2006). As shown in Figure 5, DBTCl treatment significantly reduced SOX9-positive cells in rat testis at ≥ 10 mg/kg on day 56 post-EDS treatment. Further analysis showed that DBTCl decreased SOX9 densities in individual positive-staining cells also at ≥ 10 mg/kg. These results indicate that DBTCl impairs Sertoli cell development and maturation.

### The Effects of DBTCl on Cell, Nuclear, and Cytoplasmic Size of Leydig Cells

**FIGURE 6:**
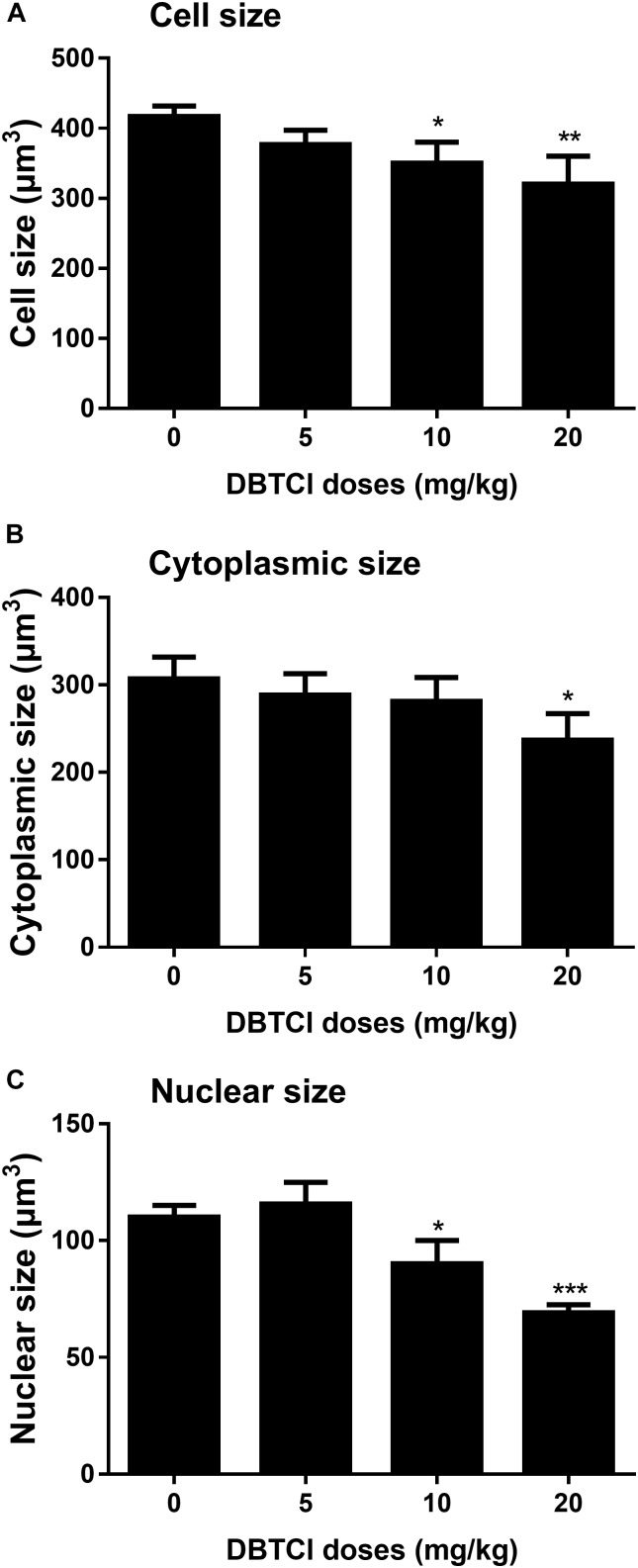
The Leydig cell size, nuclear size, and cytoplasmic size in rat testis sections from post-EDS day 56. **(A)** Leydig cell size. **(B)** Leydig cell nuclear size. **(C)** Leydig cell cytoplasmic size. Mean ± SEM, *n* = 6. ^∗^, ^∗∗^, ^∗∗∗^ indicate significant difference when compared to the control at *p* < 0.05, 0.01, and 0.001, respectively.

Cell size, nuclear size, and cytoplasmic size of Leydig cells in the testis were quantified on day 56 post-EDS treatment. It was found that DBTCl significantly reduced Leydig cell and nuclear size at ≥ 10 mg/kg (Figures 6A,C) and cytoplasmic size at 20 mg/kg (Figure 6B), indicating that DBTCl retards Leydig cell maturation.

## Discussion

Leydig cells in the testis interstitial compartment contribute 95–99% of circulatory testosterone (Wu et al., 2017). Testosterone biosynthesis is catalyzed by SCARB1, STAR, CYP11A1, 3β-HSD, CYP17A1, and 17β-HSD3 (Wang et al., 2011), which gets promoted when LH binds to LHCGR on its surface (Melsert et al., 1991; Foster, 1996; Zhang et al., 2018).

Organotin DBT is primarily used to stabilize polyvinyl chloride plastic products. Most often, it reacts with HCl and produces DBTCl (Boyer, 1989). There have been some reports of high DBTCl biological accumulation. For example, in the United Kingdom, the average DBTCl concentration in oysters was found to be 16.1 μg/g (Ebdon et al., 1989). DBTCl can also enter the human body via food consumption, as DBTCl concentration in some fish in the market was 0.674 μg/g (Tsunoda, 1993). Of the various toxicities of DBTCl, it has been reported to suppress testosterone production induced by tropic stimulation, thereby demonstrating its negative effects on Leydig cells (Nakajima et al., 2005; Brown et al., 2017; Furukawa et al., 2017; Milton et al., 2017; Zhang et al., 2018). Another organotin, tributyltin chloride (TBT), has been demonstrated to block Leydig cell developmental regeneration in the adult rat testis (Wu et al., 2017); therefore it is worth wondering whether DBTCl has the similar effects as both TBT and DBTCl are organotins. Thus, the present study was conducted to investigate the effects of DBTCl on Leydig cell developmental regeneration, using the EDS-induced rat Leydig cell developmental regeneration model.

EDS has been demonstrated to be a powerful tool in studying Leydig cell regeneration. Inducing adult male rats with a dose of 75 mg/kg of i.p., EDS specifically eliminates all the adult Leydig cells in the testes (Yan et al., 2000). The lack of Leydig cells and testosterone increased the secretion of LH and other cytokines, which in turn induced and stimulated Leydig cell regeneration (Kerr and Sharpe, 1985). The process of Leydig cell regeneration after EDS treatment in adult rat testis undergoes four distinct stages, which are similar to Leydig cell pubertal development: stem Leydig cells commit into progenitor Leydig cells by day 21, then differentiates into immature Leydig cells by day 28, and final matures into adult Leydig cells by day 56 (Guo et al., 2013).

DBTCl doses of 0, 5, 10, and 20 mg/kg were selected according to previous studies, which showed that oral administration to pregnant Wistar rats with DBTCl at doses ≥ 20 mg/kg caused teratogenicity and mortality (Ema et al., 1992; Furukawa et al., 2017). A 10-day DBTCl exposure exerted profound negative effects on regenerating Leydig cells. Through hormone analysis it was found that DBTCl reduced serum testosterone levels following the treatment of EDS even at the lowest dose of 5 mg/kg. This was achieved through multiple mechanisms. First, DBTCl downregulated the gene expression levels of steroidogenic enzymes including *Lhcgr, Scarb1, Star, Cyp11a1*, and *Hsd17b3*, which led to a reduction in their protein levels. It should be noted that gene expression changes did not occur in parallel to protein changes in this study, as at lower doses (5 or 10), for LHCGR, SCARB1, 17β-HSD3, and FSHR, the mRNA levels were unchanged but the protein levels were downregulated, and opposing changes happened in STAR, AMH, and SOX9. This might be explained by the reason that DBTCl affected transcription and translation processes. Secondly, DBTCl reduced advanced-stage Leydig cell number (immature and adult Leydig cells) as shown by a decrease in positive 11β-HSD1-staining cells. This decrease also contributed to the downregulation of steroidogenic gene and protein levels. Third, a decrease in 11β-HSD1 density in individual cells, along with a reduction in cell size and nuclear size, suggested that Leydig cell regeneration and maturation were retarded. It should be noted that in the testis, 11β-HSD1 is expressed exclusively in Leydig cells so it can be taken as Leydig cell marker (Ge and Hardy, 1998). However 11β-HSD1 density is a measurement of 11β-HSD1 enzyme level in individual Leydig cell, and 11β-HSD1 density changes do not necessarily occur in parallel to Leydig cell number changes precisely, so DBTCl significantly reduced Leydig cell number but not 11β-HSD1 density at 10 mg/kg. Delayed Leydig cell regeneration and impaired Leydig cell function were confirmed by the elevated LH levels via negative feedback. Collectively, these results discussed above led to the conclusion that DBTCl retards Leydig cell developmental regeneration process by directly acting on Leydig cells themselves.

It was also found another mechanism of DBTCl-mediated delay of Leydig cell regeneration through the impairment of Sertoli cells. Sertoli cell is also known as Leydig cell-supporting cell. FSH binds to FSHR on Sertoli cells to stimulate DHH and AMH production (Kim and Griswold, 2001; Blagosklonova et al., 2002), which promote Leydig cell development and regeneration (Sriraman et al., 2001; O’Shaughnessy et al., 2008). SOX9 serves as the biomarker for Sertoli cells and promotes its development and maturation (Foster, 1996; Koopman, 1999; Barrionuevo et al., 2006). The evidence obtained in this study showed that DBTCl impaired Sertoli cell function by downregulating the expression of FSHR and AMH at both the gene and protein levels. Sertoli cell function impairment was pinpointed by the elevation of FSH levels via negative feedback. The impairment undoubtedly retarded Leydig cell developmental regeneration. Furthermore, DBTCl treatment impaired Sertoli cell development and maturation, as SOX9 levels and densities were decreased.

Previous studies reported teratogenicity and lethality of DBTCl at doses ≥ 20 mg/kg, and the present study extended DBTCl toxicity to 5 mg/kg with regard to male steroidogenesis (Ema et al., 1992; Furukawa et al., 2017). Though no report concerning the *in vivo* effects of DBTCl on Leydig cell was available, there were indeed some *in vitro* studies. DBTCl dose-dependently suppressed testosterone production in isolated pig Leydig cells over the concentration range of 0.03–0.3 μM (Ohno et al., 2005). The mechanism, however, remains elusive. One possibility lies in decreasing intracellular cAMP levels as DBTCl has been proved to decrease intracellular cAMP levels in natural killer cells, and whether intracellular cAMP levels in Leydig cells would get decreased by DBTCl or not requires further investigation (Whalen and Loganathan, 2001). Decrease in intracellular cAMP could exert negative effects on the rate-limiting steroidogenic enzymes, STAR and CY11A1, which are tightly controlled by the cAMP-PKA signaling pathway (Foster, 1996).

Both TBT and DBTCl are organotins, and it has been demonstrated that a short-term exposure to TBT blocks Leydig cell developmental regeneration in the adult rat testis (Wu et al., 2017), from which it is speculated that DBTCl has similar effects. The present study testifies the hypothesis and finds that DBTCl retards Leydig cell developmental regeneration via downregulating steroidogenesis-related enzymes at both the gene and protein levels, inhibiting Leydig cell proliferation and impairing Sertoli cell function and development. Due to the detrimental effects of DBTCl on Leydig cell, further studies were suggested and recommendations were proposed for humans to keep away from DBTCl exposure. For example, humans should be careful when consuming food, as high DBTCl accumulation has been reported to be found in certain food items (Ebdon et al., 1989; Tsunoda, 1993).

## Author Contributions

YY conceived and designed the study, and wrote and polished the manuscript. XH performed the experiments and collected the data. TM analyzed the data and made the graphs.

## Conflict of Interest Statement

The authors declare that the research was conducted in the absence of any commercial or financial relationships that could be construed as a potential conflict of interest.
