# Supplementary material for: Dibutyltin Dichloride Retards Leydig Cell Developmental Regeneration in Adult Rat Testis
Source: Front Pharmacol. 2018 Nov 30;9:1320. doi: 10.3389/fphar.2018.01320 (PMC6283912; doi:10.3389/fphar.2018.01320)
Supplement: Supplementary file 1 [file Data_Sheet_1.PDF]

**Supplementary Table S1. Primer information**

| Primer Symbol | Gene name | Primer direction | Sequences (5'to 3') | Efficinecy rate (%) | PCR (bp) | Accession |
|---|---|---|---|---|---|---|
| Lhcgr | Luteinizing hormone receptor | Forward | CTGCGCTGTCCTGGCC | 91 | 103 | NM_012978 |
| | | Reverse | CGACCTCATTAAGTCCCCTGAA | | | |
| Scarb1 | Scavenger receptor class B, member 1 | Forward | ATGGTACTGCCGGGCAGAT | 93 | 117 | NM_031541 |
| | | Reverse | CGAACACCCTTGATTCCTGGTA | | | |
| Star | Steroidogenic acute regulatory protein | Forward | CCCAAATGTCAAGGAAATCA | 96 | 187 | NM_031558 |
| | | Reverse | AGGCATCTCCCCAAAGTG | | | |
| Cyp11a1 | Cholesterol side chain cleavage enzyme | Forward | AAGTATCCGTGATGTGGG | 93 | 127 | NM_017286 |
| | | Reverse | TCATACAGTGTCGCCTTTTCT | | | |
| Hsd3b1 | 3β-Hydroxysteroid dehydrogenase 1 | Forward | CCCTGCTCTACTGGCTTGC | 103 | 189 | NM_001007719 |
| | | Reverse | TCTGCTTGGCTTCCTCCC | | | |
| Cyp17a1 | P450 17α-hydroxylase/ 17,20-lyase | Forward | TGGCTTTCCTGGTGCACAATC | 99 | 90 | NM_012753 |
| | | Reverse | TGAAAGTTGGTGTTCGGCTGAAG | | | |
| Hsd17b3 | 17β-Hydroxysteroid dehydrogenase 3 | Forward | TGGATTGGCCATGCAGGATTG | 98 | 202 | NM_054007 |
| | | Reverse | TGAAAGTTGGTGTTCGGCTGAAG | | | |
| Hsd11b1 | 11β-Hydroxysteroid dehydrogenase 1 | Forward | CATTAGCGGTAGCCTTAG | 105 | 288 | NM_008288.2 |
| | | Reverse | GAGGTAGGCTTAGCATGAAC | | | |
| Fshr | Follicle stimulating hormone receptor | Forward | CCACAAGCCAATACAAACTAACT | 93 | 327 | NM_199237 |
| | | Reverse | CAAAAGTCCAGCCCAATACC | | | |
| Amh | Antimulerian hormone | Forward | GCCCTAACCCTTCAACCA | 104 | 82 | NM_012902 |
| | | Reverse | GGGAATCAGAGCCAAACAGA | | | |
| Dhh | Desert Hedgehog | Forward | AACCCCGACATAATCTTCA | 108 | 150 | NM_053367 |
| | | Reverse | CTCGTCCCAACCTTCAGT | | | |
| Sox9 | SRY box 9 | Forward | TGCTGAACGAGAGCGAGAAG | 94 | 160 | NM_080403.1 |
| | | Reverse | ATGTGAGTCTGTTCGGTGGC | | | |
| β-actin | Beta-Actin | Forward | CCATGAAGATCAAGATCAT | 96 | 106 | NM_001141945 |
| | | Reverse | TTGCTGATCCACATCTGCT | | | |
