# Supplementary material for: Dibutyltin Dichloride Retards Leydig Cell Developmental Regeneration in Adult Rat Testis
Source: Front Pharmacol. 2018 Nov 30;9:1320. doi: 10.3389/fphar.2018.01320 (PMC6283912; doi:10.3389/fphar.2018.01320)
Supplement: Supplementary file 2 [file Data_Sheet_2.PDF]

Supplementary Table 2. Antibodies

Antibody	Species	Vendor (City, State, catalogue)	Dilution	
			WB	HS
LHCGR	goat	Santa Cruz (Santa Cruz, CA, sc-26343)	1:1000	ND
SCARB1	rabbit	Abcam (San Francisco, CA, ab52629)	1:1000	ND
STAR	goat	Santa Cruz (Santa Cruz, CA, sc-23492)	1:1000	ND
CYP11A1	rabbit	Santa Cruz (Santa Cruz, CA, sc-18043)	1:1000	ND
3β-HSD1	rabbit	Abcam (San Francisco, CA, ab65156)	1:2000	1:1000
CYP17A1	rabbit	Santa Cruz (Santa Cruz, CA, sc-66850)	1:1000	ND
17β-HSD3	rabbit	Santa Cruz (Santa Cruz, CA, sc-67344)	1:2000	ND
11β-HSD1	rabbit	Abcam (San Francisco, CA, ab39364)	1:2000	1:1000
FSHR	rabbit	Abcam (San Francisco, CA, ab103874)	1:2000	ND
DHH	rabbit	Proteintech (Rosemont, IL, 13889-1-AP)	1:2000	ND
AMH	rabbit	Abcam (San Francisco, CA, ab84952)	1:2000	ND
SOX9	rabbit	Abcam (San Francisco, CA, ab3697)	1:2000	1:1000
β-actin	rabbit	Cell Signaling Technology (Danvers, MA,12620)	1:1000	ND

ND = Not detected; WB = Western blot; HS = Histochemical staining.
